# Tandem Heterogeneous Catalysis for Polyethylene Depolymerization via an Olefin-Intermediate Process

**DOI:** 10.1021/acssuschemeng.0c07612

**Published:** 2021-01-08

**Authors:** Lucas D. Ellis, Sara V. Orski, Grace A. Kenlaw, Andrew G. Norman, Kathryn L. Beers, Yuriy Román-Leshkov, Gregg T. Beckham

**Affiliations:** † Renewable Resources and Enabling Sciences Center, National Renewable Energy Laboratory, Golden, Colorado 80401, United States; ‡ Materials Science and Engineering Division, National Institute of Standards and Technology, Gaithersburg, Maryland 20899, United States; ¶ Materials Science Center, National Renewable Energy Laboratory, Golden, Colorado 80401, United States; § Department of Chemical Engineering, Massachusetts Institute of Technology, Cambridge, Massachusetts 02139, United States

**Keywords:** Olefin-intermediate process, Polyethylene, Plastics upcycling, Olefin cross metathesis, Dehydrogenation

## Abstract

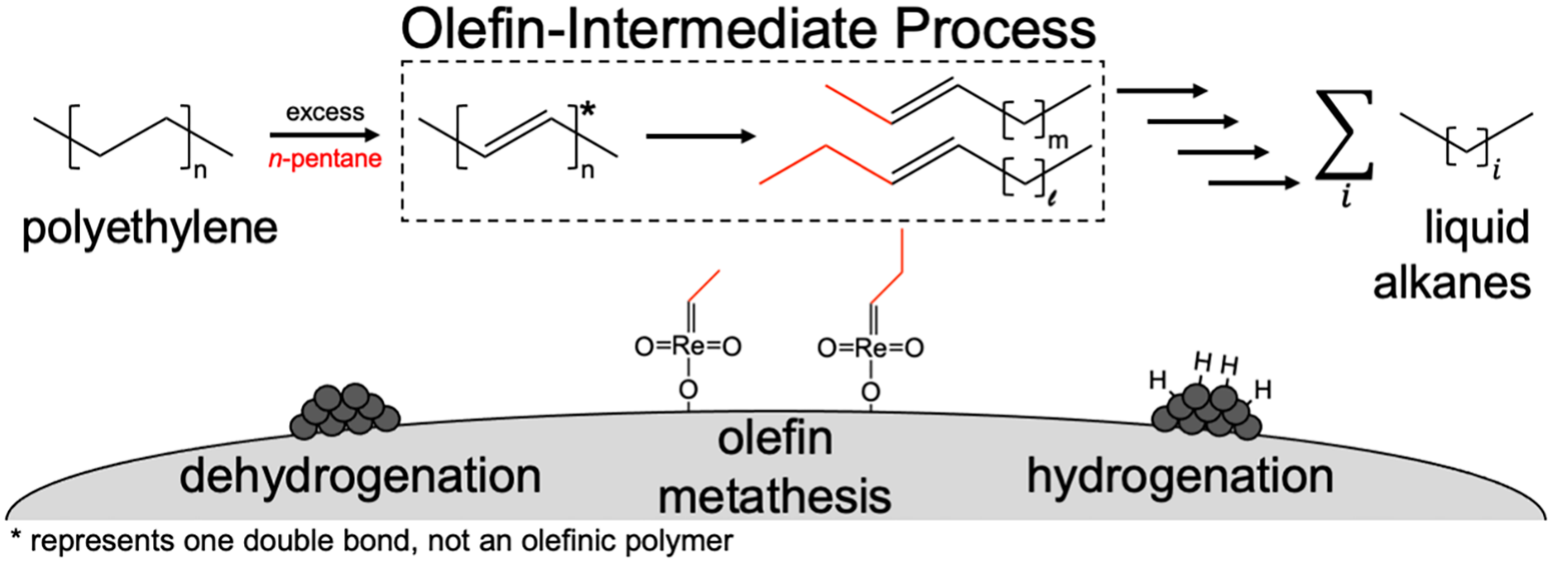

The accumulation of plastic waste in the environment has prompted the development of new chemical recycling technologies. A recently reported approach employed homogeneous organometallic catalysts for tandem dehydrogenation and olefin cross metathesis to depolymerize polyethylene (PE) feedstocks to a mixture of alkane products. Here, we build on that prior work by developing a fully heterogeneous catalyst system using a physical mixture of SnPt/γ-Al_2_O_3_ and Re_2_O_7_/γ-Al_2_O_3_. This heterogeneous catalyst system produces a distribution of linear alkane products from a model, linear C_20_ alkane, *n*-eicosane, and from a linear PE substrate (which is representative of high-density polyethylene), both in an *n*-pentane solvent. For the PE substrate, a molecular weight decrease of 73% was observed at 200 °C in 15 h. This type of tandem chemistry is an example of an olefin-intermediate process, in which poorly reactive aliphatic substrates are first activated through dehydrogenation and then functionalized or cleaved by a highly-active olefin catalyst. Olefin-intermediate processes like that examined here offer both a selective and versatile means to depolymerize polyolefins at lower severity than traditional pyrolysis or cracking conditions.

## Abstract 2

A heterogeneous catalyst system was developed to depolymerize polyethylene to a mixture of linear alkanes at temperatures ≤200°C.

## Introduction

Nearly 60% of the 8.3 billion metric tons of plastics produced between 1950 and 2015 were landfilled or leaked into the environment, with only 7% having been recycled.^1^ Additionally, the life-cycle greenhouse gas (GHG) emissions for conventional plastics manufacturing in 2015 alone was 1.8 GtCO_2_e, corresponding to 3.8% of global emissions.^2^ Thus, there is a substantial global need to develop new recycling technologies to incentivize the recycling of plastic wastes.^3−7^ By creating diverse, catalysis-enabled processes capable of producing chemicals from waste plastics, chemical recycling could offer a means to improve plastics waste management.

**Scheme 1:**
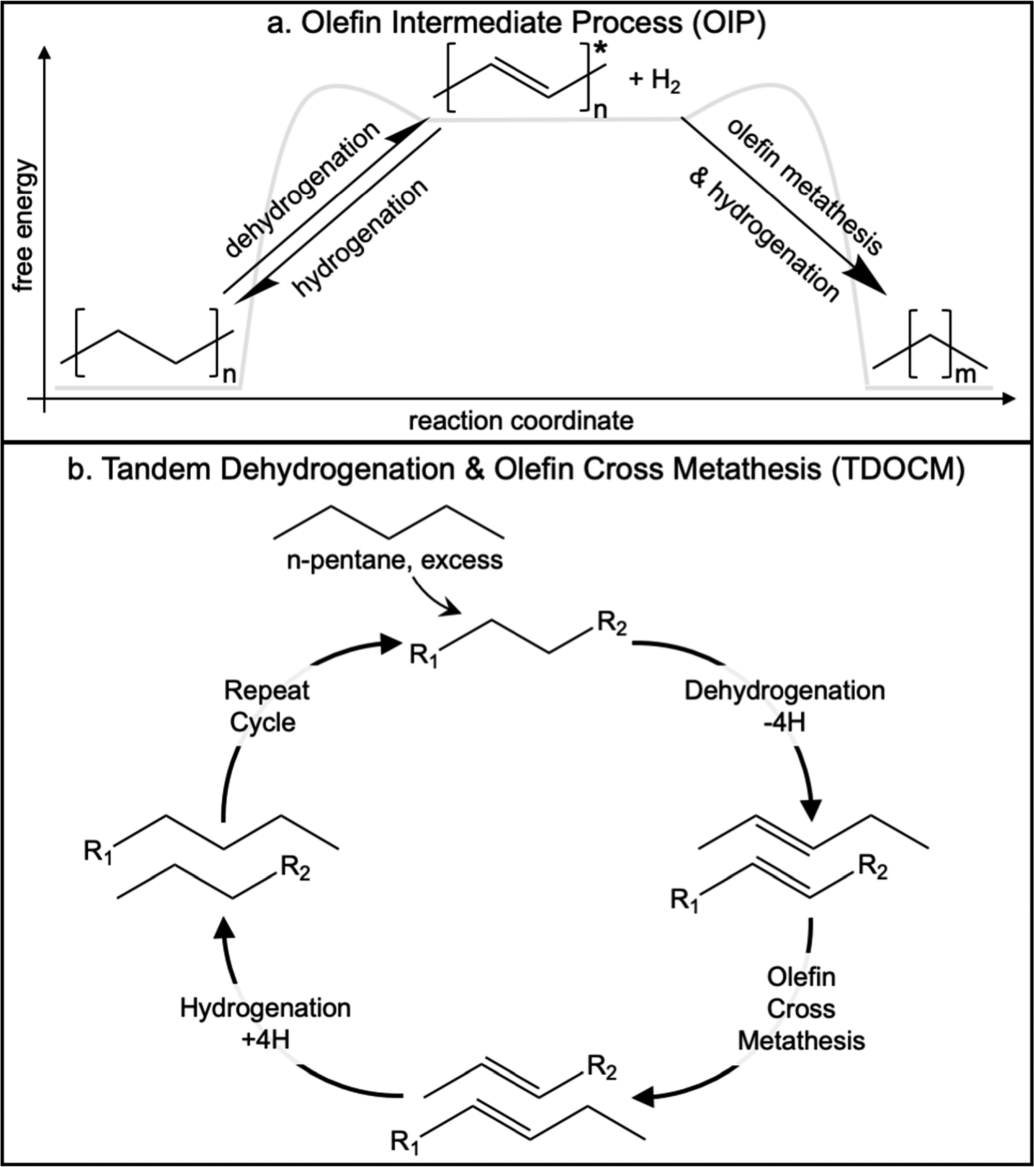
(a) OIP and (b) TDOCM of a Generic Alkane and *n*-Pentane Results in a Distribution of Alkane Products An asterisk (*) represents an olefin formed in the polymer backbone, not a conjugated olefinic polymer.

The most abundant polymer produced today is polyethylene (PE), which accounts for 25% of global plastics production.^9^ Catalytic approaches for PE depolymerization generally begin with C–H activation and can proceed via a variety of reaction intermediates, such as a carbocation for catalytic cracking,^10^ a carbon-centered radical for free-radical processes like oxidation,^11,12^ an adsorbed intermediate for hydrogenolysis,^13−15^ or a stable olefin for novel strategies like tandem dehydrogenation and olefin cross metathesis (abbreviated here as TDOCM).^16,17^ While catalytic cracking of PE typically results in mixtures of olefins or aromatic products,^10,18^ and free radical oxidation results in carboxylic acids, aldehydes, and other oxygenates,^11,12^ hydrogenolysis^13,19^ and TDOCM^17^ technologies are capable of depolymerizing PE to a distribution of alkane products. TDOCM is an example of an olefin-intermediate process (OIP) (Scheme 1a), which relies on a highly-active catalytic reaction coupled with the equilibrium-limited process of C–H dehydrogenation to drive the reaction. This strategy is similar to that used by many biological catalysts featuring a series of sequential reactions that consume the products of poorly favorable reactions to “pull” reactants through thermodynamically limited reaction steps.

TDOCM first gained attention as an approach to achieve the versatility of olefin metathesis rearrangements using alkane substrates at relatively mild conditions.^16^ In 2006, Goldman et al. utilized catalysts like a homogeneous iridium complex and a Schrock-type metathesis catalyst to perform selective dehydrogenation followed by metathesis to rearrange the chemical functionalities of two olefins.^16^ The hydrogen atoms abstracted in the dehydrogenation step were subsequently reincorporated into the olefin intermediates, resulting in a rearranged mixture of alkane products (Scheme 1b). Goldman et al. demonstrated this concept with *n*-decane (and *n*-hexane) self-rearrangement, producing a distribution of alkane products ranging from C_2_–C_30_ from reactions conducted up to 9 days.^16^ More recently, TDOCM was used to depolymerize a PE feedstock by crossing a short alkane with the long polymer backbone. Specifically, Jia et al. utilized various homogeneous iridium complexes for dehydrogenation with a heterogeneous olefin metathesis catalyst (Re_2_O_7_/γ-Al_2_O_3_) to depolymerize PE to a distribution of liquid alkane products and waxy solids, using *n*-octane or *n*-hexane as both the solvent and coreactant.^17^ The work from Jia et al. also sought to improve recoverability of the iridium complex by covalently tethering the catalyst to a support.^17^ Additionally, nearly 50 years ago, researchers at Chevron Corporation reported a heterogeneous Pt and W system that appeared to perform dehydrogenation and olefin metathesis of butane but operated at temperatures between 343 °C and 427 °C, a temperature range that approaches the operating conditions for some catalytic cracking processes.^20,21^ To date, no fully heterogeneous TDOCM catalyst system has been reported for polyolefin depolymerization to our knowledge.

Here, we developed a fully heterogeneous catalyst system for PE deconstruction via tandem dehydrogenation (Pt/γ-Al_2_O_3_, SnPt/γ-Al_2_O_3_) and olefin metathesis (Re_2_O_7_/γ-Al_2_O_3_). Noble metals such as Pt are well-known heterogeneous catalysts for C–H activation.^22^ However, nonoxidative alkane dehydrogenation is an equilibrium-limited reaction that requires temperatures well above 400 °C and low pressures to achieve high alkane conversions,^22^ while the active sites of the Re-based heterogeneous olefin metathesis catalysts are unstable above 100 °C.^23−26^ Thus, a challenge for this system is how to kinetically couple both reactions such that the system features both high activity and stability.

## Results and Discussion

**Figure 1:**
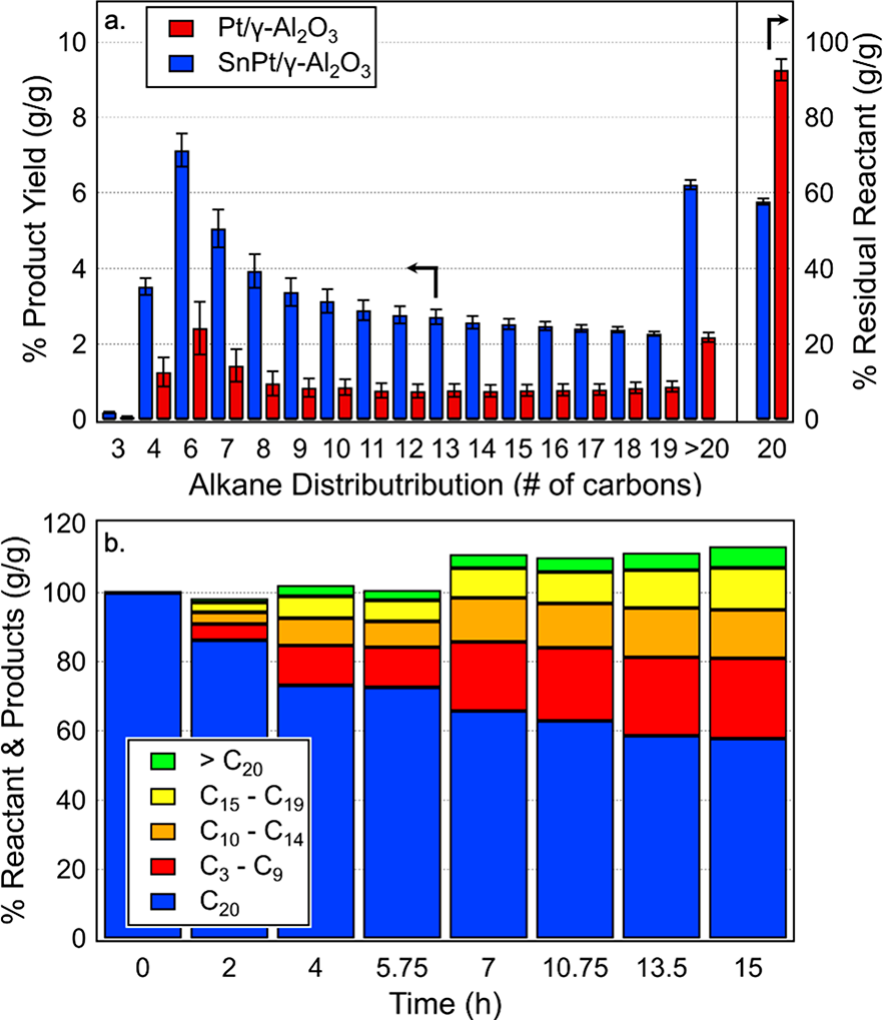
TDOCM of *n*-pentane and 5% (g/g) *n*-eicosane at 200 °C resulting in (a) a distribution of linear alkane products from a reaction of 500 mg of Re_2_O_7_/γ-Al_2_O_3_ and 500 mg of either Pt/γ-Al_2_O_3_ or SnPt/γ-Al_2_O_3_ for 15 h and (b) a time-course with 500 mg of Re_2_O_7_/γ-Al_2_O_3_ and 500 mg of SnPt/γ-Al_2_O_3_. Time-series data represent a single reaction measurement from a sacrificial reactor. End point analysis (*t* = 15 h) data are shown as the average of reaction duplicates with error bars shown as ±^1^/_2_ the range.

We first developed a pretreatment process for preparing the olefin metathesis catalyst for performance testing in 75 mL batch reactors using the coupling of 1-octene to 7-tetradecene as a model reaction (Figures S1 and S2). After developing the catalyst pretreatment system, we investigated the ideal temperature range for TDOCM using the alkane rearrangement of 5% (g/g) *n*-eicosane (*n*-C_20_H_42_) in *n*-pentane with a 1:1 physical mixture of 8% Re/γ-Al_2_O_3_ (referred to hereafter as Re_2_O_7_/γ-Al_2_O_3_) and 1.7% Sn and 0.8% Pt/γ-Al_2_O_3_ (referred to hereafter as SnPt/γ-Al_2_O_3_); these experiments demonstrated that 200 °C provided the highest conversion in the temperature range studied (Figure S3). We then compared the activity of a 1:1 physical mixture of Re_2_O_7_/γ-Al_2_O_3_ and commercially available 5% Pt/γ-Al_2_O_3_ (referred to as Pt/γ-Al_2_O_3_ for simplicity). After 15 h at 200 °C, the system with Pt/γ-Al_2_O_3_ provided a *n*-eicosane conversion of 6.3% ± 0.8%, generating a product distribution centered around the solvent, *n*-pentane (Figure 1a). The products from this reaction are a distribution of linear alkanes (Figure 1a) from C_3_ to C_35_ with the most prevalent being *n*-hexane, *n*-heptane, and *n*-butane, respectively. The 1:1 physical mixture of Re_2_O_7_/γ-Al_2_O_3_ and SnPt/γ-Al_2_O_3_ demonstrated a significant improvement in activity with a conversion of 41.6% ± 0.4% under identical conditions. The reaction products from the SnPt/γ-Al_2_O_3_ system exhibit a similar distribution in product selectivity to the Pt/γ-Al_2_O_3_ system, again centered around *n*-pentane, with the most prevalent measured products being *n*-hexane, *n*-heptane, and *n*-butane, respectively. Notably, the SnPt/γ-Al_2_O_3_ catalyst exhibits a reactive surface area that was 48% that of Pt/γ-Al_2_O_3_ (Table S1), suggesting a nearly 14-fold higher rate of *n*-eicosane disappearance per reactive surface area compared to the system with Pt/γ-Al_2_O_3_ catalyst. Tin is known to act as a dehydrogenation promoter for Pt, due, in part, to reduced overall deactivation rates.^22^ However, comparing the 5%Pt/γ-Al_2_O_3_ to a synthesized Pt/γ-Al_2_O_3_ of similar Pt loading and using the same γ-Al_2_O_3_ support as the SnPt/γ-Al_2_O_3_, in a 1:1 physical mixture with Re_2_O_7_/γ-Al_2_O_3_, resulted in an *n*-eicosane conversion of 39.1% ± 3.0% at identical reaction conditions (Figure S4). This suggests the ensemble effect of the SnPt/γ-Al_2_O_3_ may not play as significant a role as other effects, such as particle size. Regardless, future work will focus on fully characterizing the structure–property relations of the two catalysts in this tandem chemistry, considering the apparent complexity. Control reactions using Pt/γ-Al_2_O_3_, Re_2_O_7_/γ-Al_2_O_3_, or SnPt/γ-Al_2_O_3_ alone in the conversion of 5% (g/g) *n*-eicosane in *n*-pentane exhibited no measurable activity (Figure S5).

To track catalyst activity as a function of time, we collected a time series of the TDOCM reaction of *n*-pentane and 5% (g/g) *n*-eicosane with SnPt/γ-Al_2_O_3_ and Re_2_O_7_/γ-Al_2_O_3_ at 200 °C. The catalyst system deactivated after 15 h (Figure 1b) after approximately 225 turnovers (based on the number of dehydrogenation sites). Dehydrogenation catalysts are known to deactivate over time partly due to formation of carbonaceous deposits,^27,28^ and nonoxidative dehydrogenation often requires unit operations for catalyst regeneration with oxygen and halides (e.g., Cl), to remove coke and prevent sintering.^29^ The postreaction catalyst was significantly darker in color as compared to the starting material (Figure S6) and showed a 49% reduction in reactive surface area (on a dehydrogenation catalyst basis) compared with the fresh catalyst (Table S1), suggesting carbonaceous deposits did reduce the available active surface area. Additionally, it is also possible that the active sites of the rhenium olefin metathesis catalyst deactivate, because this catalyst is known to decompose over time as well.^23−26^ Regardless, the total recovered linear alkane products is greater than the consumed *n*-eicosane reactant (Figure 1b), which is expected, because the solvent is a reactant in this chemistry. Thus, a mass balance of products and residual reactants is greater than unity, because some of the carbon from the products originates from the solvent. We measured a total carbon balance from the starting *n*-eicosane of 110% and 113% for the system with Pt/γ-Al_2_O_3_ or SnPt/γ-Al_2_O_3_, respectively (Figure S7). This highlights a challenge in this chemistry, namely, to design a catalyst system that favors cross reactions of the reactant and solvent over reactant/reactant or solvent/solvent reactions.

We were interested in studying the effects of catalyst before and after pretreatment. Interestingly, we observed that the catalysts that were physically mixed before pretreatment demonstrated more than double the *n*-eicosane alkane rearrangement activity compared to the catalysts that were physically separated during pretreatment (Figure S8). To gain insights into this observation, we utilized scanning transmission electron microscopy with energy dispersive x-ray spectroscopy (STEM-EDS) to map the elemental composition of the catalysts pretreated separately or comingled. The catalysts that were separate during pretreatment exhibited the expected elemental composition, such that no Sn or Pt was found on the Re_2_O_7_/γ-Al_2_O_3_ catalyst that was pretreated by itself and no Re was found on the SnPt/γ-Al_2_O_3_. However, when the catalysts were comingled during pretreatment, six out of eight particles analyzed using STEM-EDS contained all three elements, Sn, Pt, and Re, even though these elements were synthesized on separate supports, and the remaining two out of eight particles contained both Sn and Re (Figures S9 and S10). This finding is not entirely surprising, considering the melting point of bulk Re_2_O_7_ is 297 °C and the pretreatment was conducted at 500 °C.^26^ Additionally, Pt is well-known to migrate in high temperature oxidation conditions through processes like Ostwald ripening or particle migration and coalescence.^30^ Because pretreating the catalysts in a mixed state enhanced the activity of *n*-eicosane rearrangement, and the elements of Pt, Sn, and Re were found on single supports, there could be synergistic activity due to physical proximity of the two reaction distinct reaction sites, or promotion of catalytic activity with the creation of a newly doped alloy. In the case of dehydrogenation catalysts, rhenium is known to be a promoter and has been studied as a dopant for enhancing dehydrogenation activity.^31,32^

Based on these results, we synthesized a catalyst with both chemical functionalities on one support to determine whether a single supported catalyst could perform as well as a physical mixture of the two catalysts. We synthesized both Re_2_O_7_ on SnPt/γ-Al_2_O_3_ or SnPt on Re_2_O_7_/γ-Al_2_O_3_ through alternating sequential incipient wetness (i.e., via an initial synthesis of SnPt/γ-Al_2_O_3_, then via deposition of Re_2_O_7_ in another round of incipient wetness, or vice versa). Both catalysts exhibited a slight increase in overall dehydrogenation reactive surface area, as compared to the native SnPt, thus the following analysis has roughly equivalent dehydrogenation sites (Table S1). Surprisingly, both catalysts were poor performers, with *n*-eicosane conversion of 10.8% ± 8.0% and 7.0% ± 4.1%, respectively, at 200 °C in 15 h. Both of these results are significantly lower compared to the 41.6% ± 0.4% conversion obtained with the physical mixture of Re_2_O_7_/γ-Al_2_O_3_ on SnPt/γ-Al_2_O_3_ (Figure S11). It is known that the Re olefin metathesis active site is strongly influenced by the support;^26^ thus, Re deposited on bulk Pt or SnPt, as an alloy, would not likely be active in olefin cross metathesis. We hypothesized that formation of alloys with Pt or SnPt reduced the population of active metathesis sites because alloys of PtRe, PtSn, or ReSn (ignoring alkylation) have not been reported to exhibit olefin metathesis activity to our knowledge. We tested this hypothesis by adding additional γ-Al_2_O_3_ support, physically mixed with the Re_2_O_7_ on SnPt/γ-Al_2_O_3_ or SnPt on Re_2_O_7_/γ-Al_2_O_3_, with the expectation that pretreatment would allow for migration of the Re on the catalyst to the new support, forming olefin cross metathesis sites. The addition of support increased the *n*-eicosane conversion to 18.8% ± 1.5% and 10.9% ± 3.9%, respectively. Lastly, we supplemented Re_2_O_7_ on SnPt/γ-Al_2_O_3_ or SnPt on Re_2_O_7_/γ-Al_2_O_3_ with additional Re_2_O_7_/γ-Al_2_O_3_. All cases showed that supplementation by additional Re_2_O_7_/γ-Al_2_O_3_ to either Re_2_O_7_ on SnPt/γ-Al_2_O_3_ or SnPt on Re_2_O_7_/γ-Al_2_O_3_ provided the highest conversion of *n*-eicosane, within error of a physical mixture of Re_2_O_7_/γ-Al_2_O_3_ and SnPt/γ-Al_2_O_3_. This result implies that the population of olefin metathesis sites is lower on the single support, compared with a physical mixture of the catalysts on two separate supports.

**Figure 2:**
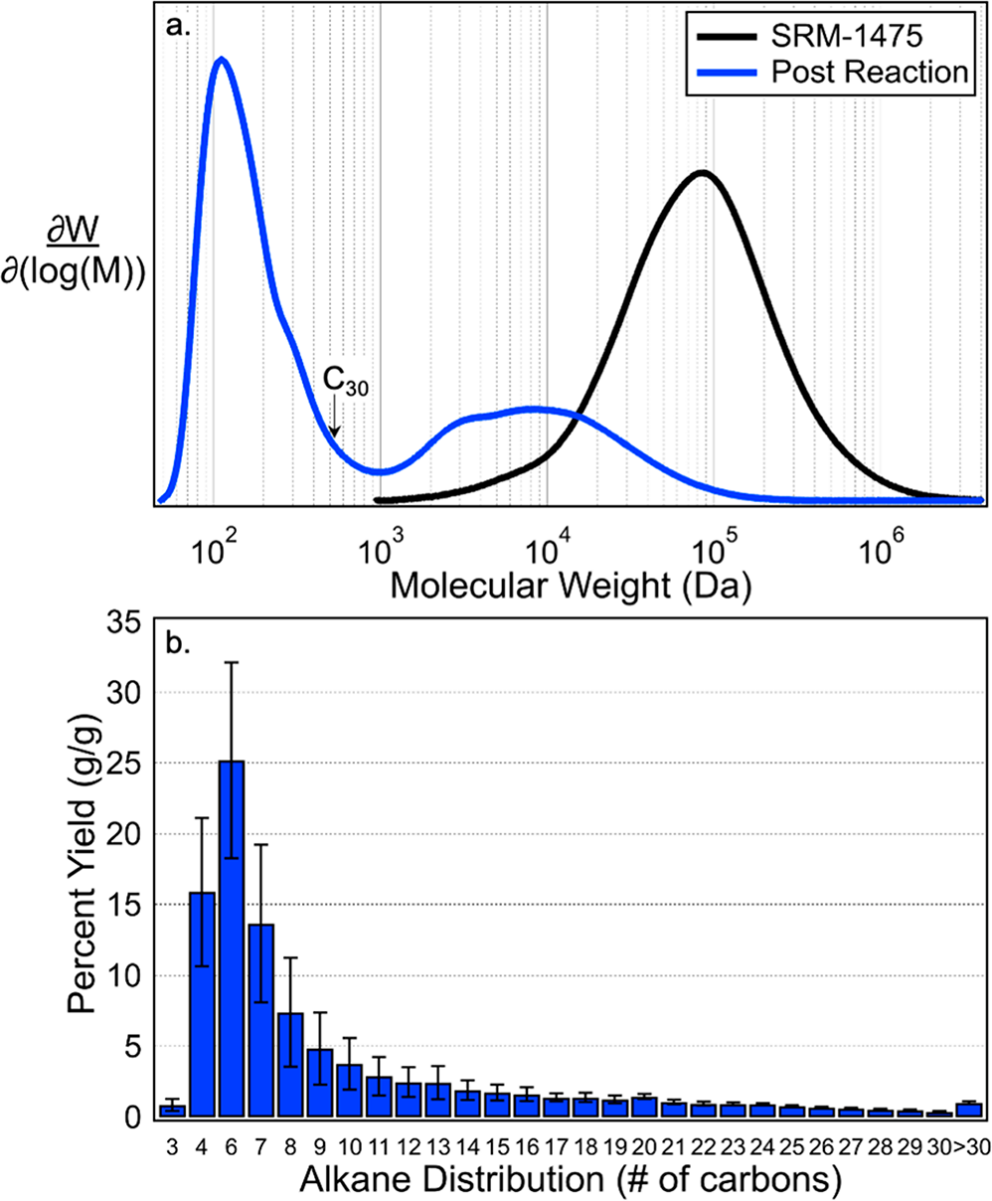
(a) The molecular weight distribution of the native high-density PE feedstock (SRM-1475) and post reaction solids and (b) the product yield of the distribution of alkane products from the depolymerization of 130 mg of SRM-1475 PE feedstock in *n*-pentane. The product distribution is the average of three reaction replicates, and the error bars represent the standard deviation.

Lastly, we sought to utilize this system to depolymerize a standard reference material (SRM) from the National Institute for Standards and Technology (NIST) for linear PE, SRM-1475.^33−42^ This linear PE feedstock is a standard reference material for high-density PE and has a measured molecular weight of 54.1 ± 2 kDa in our study (Figure 2). We first explored the effect of reaction temperature on alkane product yield. The highest yield of alkane products occurred at 200 °C (Figure S12). We then ran three reaction replicates at 200 °C (Figure 2) and measured the depolymerization extent with high-temperature gel permeation chromatography. Comparing the molecular weight distribution for the original polymer and the degraded SRM-1475, while ignoring compounds below a molecular weight of 500 Da, a 73% reduction in average molecular weight was obtained. Figure 2 summarizes the distribution of measured products and the reduction in molecular weight. The total yield (g/g) of recovered alkanes in the liquid phase from the 130 mg loading of PE was 99%; additionally, the residual polymer had a molecular weight that was 27% of the starting material (Figure 2a), suggesting a carbon balance of 126% (Figure S13). In a control reaction with no polymer and only solvent and catalyst present, no alkanes longer than C_13_ were measured, suggesting larger products are derived from the polymer (Figure S14). Further studies will require isotopic tracking to quantitatively characterize the level of solvent/solvent, solvent/polymer, and possibly, polymer/polymer reactions.

## Conclusions

We demonstrated that a heterogeneous dehydrogenation catalyst and a heterogeneous olefin metathesis catalyst are capable of alkane rearrangement for liquid alkanes or PE in *n*-pentane, both resulting in a distribution of *n*-alkane products. This work provides a foundation for future studies to probe the mechanism, kinetics, desirable process configurations, and the ideal structure–function properties for catalysts to provide for maximal rates and tunable product distributions. Future fundamental studies are needed to understand the amount of undesired solvent–solvent reactions compared with desired solvent-feedstock reactions. Additionally, this study employed high-loadings of expensive noble metal catalysts; thus, more effort is required to lower catalyst loadings, understand the regeneration and recycling of such catalysts, and if possible, explore whether inexpensive metals are capable of such chemistry to minimize catalyst costs. Lastly, to enhance the profitability of such a process, alternative solvents should also be explored to possibly provide for alternative and higher value products. More broadly, we believe TDOCM is one of many future examples of an OIP capable of deconstruction of C–C bonded waste plastics.

## Supporting Information Available

Experimental methods, catalyst preparation equipment, reaction results, and catalyst characterization data (PDF)

## Abbreviations

TDOCM: tandem dehydrogenation and olefin cross metathesis
OIP: olefin-intermediate process
PE: polyethylene
Pt/γ-Al_2_O_3_: 5% Pt/γ-Al_2_O_3_, Sigma-Aldrich
SnPt/γ-Al_2_O_3_: 1.7% Sn 0.8% Pt/γ-Al_2_O_3_, synthesized, incipient wetness
Re_2_O_7_/γ-Al_2_O_3_: 8% Re/γ-Al_2_O_3_, synthesized, incipient wetness
SRM-1475: Standard Reference Material-1475, PE feedstock with *M*_w_ = 54.1 ± 2 kDa
