# Supplementary material for: Tandem Heterogeneous Catalysis for Polyethylene Depolymerization via an Olefin-Intermediate Process
Source: ACS Sustain Chem Eng. 2021 Jan 8;9(2):623–8. doi: 10.1021/acssuschemeng.0c07612 (PMC11066966; doi:10.1021/acssuschemeng.0c07612)
Supplement: Supplementary file 1 — sc0c07612_si_001.pdf [file sc0c07612_si_001.pdf]

Supporting Information for:

Tandem heterogeneous catalysis for polyethylene depolymerization via an olefin-intermediate process

Lucas D. Ellis,[†] Sara V. Orski,[‡] Grace A. Kenlaw, [‡] Andrew G. Norman,[¶] Kathryn L. Beers,[‡] Yuriy Román-Leshkov,*[§] and Gregg T. Beckham*[†]

[†] Renewable Resources and Enabling Sciences Center, National Renewable Energy Laboratory, Golden, CO 80401, USA
[‡] Materials Science and Engineering Division, National Institute of Standards and Technology, Gaithersburg, MD 20899, USA
[¶] Materials Science Center, National Renewable Energy Laboratory, Golden, CO 80401, USA
[§] Department of Chemical Engineering, Massachusetts Institute of Technology, Cambridge, MA 02139, USA
* Corresponding Author E-mails:
gregg.beckham@nrel.gov
yroman@mit.edu

Number of pages: 22

Number of Figures: 12

Number of Tables: 1

Supporting Information Content **page**

Supporting Information Content	page
**Abbreviations**	**S2**
**Experimental Methods**	**S3-S5**
**Figure S1**	**S6**
**Figure S2**	**S7**
**Figure S3**	**S8**
**Table S1**	**S9**
**Figure S4**	**S10**
**Figure S5**	**S11**
**Figure S6**	**S12**
**Figure S7**	**S13**
**Figure S8**	**S14**
**Figure S9**	**S15**
**Figure S10**	**S16**
**Figure S11**	**S17**
**Figure S12**	**S18**

References S19

ABBREVIATIONS

- TDOCM – tandem dehydrogenation and olefin cross metathesis
- OIP – olefin-intermediate process
- Pt/γ-Al_2O_3 – 5% Pt/γ-Al_2O_3, Sigma Aldrich (details below)
- SnPt/γ-Al_2O_3 – 1.7% Sn 0.8% Pt/γ-Al_2O_3, synthesized, incipient wetness (details below)
- Re_2O_7/γ-Al_2O_3 – 8% Re/γ-Al_2O_3, synthesized, incipient wetness (details below)
- SRM-1475 – standard reference material-1475, polyethylene feedstock with $M_w = 54.1 \pm 2$ kDa
- PE – polyethylene
- UHP – ultra-high purity
- HT-SEC – high-temperature size exclusion chromatography
- RI – refractive index
- TEM – transmission electron microscopy
- STEM-EDS – scanning transmission electron microscopy with energy dispersive X-ray spectroscopy
- GC-FID – gas chromatography with a flame ionization detector
- ICP-OES – inductively coupled plasma - optical emission spectrometry
- BET – Brunauer-Emmett-Teller
- GC-MS – gas chromatography mass spectrometry
- g – gram

EXPERIMENTAL METHODS

Catalyst Synthesis

Catalysts were synthesized using incipient wetness on a γ-Al_2O_3 support (CAS: 1344-28-1, Strem Chemicals Inc., Newbury, MA, USA). Platinum on alumina (Pt/γ-Al_2O_3) at 5% (g/g) loading (Pt basis) was purchased from Sigma Aldrich (MDL: MFCD00011179, St. Louis, MO, USA). Supported Re_2O_7/γ-Al_2O_3 at an 8% (g/g) loading (Re basis) was synthesized using 75-80% perrhenic acid solution (CAS: 13768-11-1, Sigma Aldrich, St. Louis, MO, USA) with deionized (DI) water as the solvent. Supported SnPt/γ-Al_2O_3 with a 1.7% (g/g) Sn and 0.8% (g/g) Pt loading (Sn and Pt basis) utilized a sequential deposition, first 98% tin (II) chloride (CAS: 10025-69-1, Sigma Aldrich, St. Louis, MO, USA) was dissolved in 200 proof ethanol, mixed with the support, and calcined (temperature profile below). Subsequently, the material was functionalized with Pt using 99.9% chloroplatinic acid hydrate (CAS: 26023-84-7, Sigma Aldrich, St. Louis, MO, USA). The 0.8% Pt catalyst was synthesized with platinum nitrate dihydrate (CAS: 32916-07-7, Sigma Aldrich, St. Louis, MO, USA). All impregnated supports were calcined in air with a temperature profile of 2 °C/min from room temperature to 120 °C, held for 4 h, followed by a ramp of 5 °C/min to 550 °C and held for 6 hours.

Catalyst Performance Testing

Reactions were performed on a Series 5000 Multiple Reactor System by Parr Instruments (Moline, IL, USA), allowing for six 75 mL total volume reactors to be run in parallel with temperature control and magnetic mixing. These reactors were filled with 30 mL of reaction medium (either solvent only in the case of solid substrates or solvent and soluble reactant in the case of *n*-eicosane). Prior to preparing solutions for reactors, all glassware was dried in 100 °C oven. For the soluble reactant, ~6.33 g of 99% *n*-eicosane (CAS: 112-95-8, ACROS Organics, Fair Lawn, NJ, USA) was added to a volumetric flash and brought up to 200 mL with +99%, anhydrous, *n*-pentane (CAS: 109-66-0, ACROS Organics, Fair Lawn, NJ, USA). This solution was then transferred to a round bottom flask and dried with the addition of 2.0 g of pre-dried molecular sieves, sealed with a rubber stopper and purged with ultra-high purity (UHP) argon. A 30 mL volume was then added to each reactor, while each reactor continuously purged with UHP argon. A sample of this solution was retained as a pre-reaction mixture for determination of conversion. Mass balances of the reactors were performed to track the exact amount of solution added and any losses of *n*-pentane due to evaporation. Once reactors were prepared with reaction solution, catalysts were then pretreated.

Catalysts were prepared for reaction using a custom pretreatment reactor system (**Figure S1**). Half-inch Swagelok tube fittings were selected with a large steel mass to ensure higher heat capacity during the transition periods of the pretreatment process (**Figure S1**). The tubes were layered sequentially with 300 mg of packed quartz wool, 200 mg of γ-Al_2O_3, the mixed catalyst, 200 mg of γ-Al_2O_3, and covered in perforated aluminum foil. The tubes were then heated in a muffle furnace under atmosphere at a ramp rate of 12 °C/min to 500 °C and held for 1 hour. Tubes were then rapidly transferred (in a hot state) to an UHP argon (Airgas, Radnor, PA, USA) purge system (**Figure S1**). Only three tubes were brought at a time to the UHP argon purge, leaving the other three in the muffle furnace during this transition. The first three tubes were loaded on the purge system, argon flow started, and monitored to ensure adequate flow. Once installed and purging, the last three tubes then went through the same process. Tubes were allowed to purge for 15 min, until they reached near ambient temperature. Individual reactors were prepared prior to catalyst pretreatment, with solvent, magnetic stir bar, and soluble reactant (i.e. *n*-eicosane where relevant). A UHP argon gas line was then connected to the head of a Parr reactor and flow initiated. Pretreatment tubes were then removed from the purge apparatus and rapidly evacuated into a Parr reactor, solid substrates (i.e. PE where relevant) was then added, and the reactor was sealed under flowing UHP Argon.

The Series 5000 Multiple Reactor System by Parr Instruments has temperature control with a ~30 minutes transient heat-up to reach reaction temperature and magnetic mixing. All reported reaction times (e.g. 15 h) were for total duration from ambient temperature, meaning the first 30 minutes of the reaction is the transient heat up to the desired reaction temperature. Reactors were quenched in an ice bath at the end of reaction testing. Reactors were purged with UHP helium (Airgas, Radnor, PA, USA) a total of three times

prior to initiating temperature control. The third pressurization was used as a leak test to ensure the reactors had an adequate seal. The final pressure was set to 40 bar He prior to heating. We tested whether reaction performance was dependent on helium pressure by running tandem dehydrogenation and olefin cross metathesis (TDOCM) of *n*-pentane and *n*-eicosane at 20 bar and 40 bar He. The conversion of 5% (g/g) *n*-eicosane in *n*-pentane at 200 °C for 15 hours with a physical mixture of 500 mg Re_2O_7/γ-Al_2O_3 and 500 mg of SnPt/γ-Al_2O_3 at 20 bar He or 40 bar He, was 40.5%±1.7% (g/g) and 41.6%±0.4% (g/g), respectively, where the error was measured as half the range of duplicates. All subsequent reactions were run using 40 bar He. All reaction data were run in reaction duplicate, and the average of two measurements is reported with the error representing ±½ the range, unless otherwise stated.

Post reaction, a well-mixed liquid sample was filtered with a 0.2 μm syringe filter. Liquid samples were analyzed by injection on an Agilent 6890N gas chromatograph with a flame ionization detector (GC-FID) and equipped with a HP-5MS column (30 m x 0.250 mm x 0.25 μm). Chromatographic separation was achieved using the following GC oven program: 50 °C (hold 1 min), 15 °C/min to 180 °C (hold 1 min), ramp 20 °C/min to 325 °C (hold 6 min). A 1 mL volume was injected into the inlet, which was set to 300°C. UHP helium (Airgas, Radnor, PA, USA) was used as the carrier gas for the system. An alkane standard mixture of linear alkanes from C_8-C_{40} (AccuStandard, New Haven, CT, Part number: PS-CP-06A-1ML) was used for quantification of products. Internal standard calibration curves were generated by diluting the standard mix with *n*-pentane and spiking with mesitylene (CAS: 108-67-8, ACROS Organics, Fair Lawn, NJ, USA) as the internal standard. A *n*-heptane (CAS: 142-82-5, ACROS Organics, Fair Lawn, NJ, USA) calibration curve was generated, in-house, in *n*-pentane (CAS: 109-66-0, ACROS Organics, Fair Lawn, NJ, USA). All other compounds utilized effective carbon number to determine a response factor. Conversion of *n*-eicosane was determined by first using the concentration of *n*-eicosane determined in the pre-reaction solution and the mass of solution added to each reactor, to calculate the initial mass of *n*-eicosane, and the final mass of each solution with the concentration determined via GC. Thus, conversion was defined as = ($C_{20}^{Initial}$ – C_{20}^{Final})/ $C_{20}^{Initial}$, were $C_{20}^{Initial}$ = the original mass of *n*-eicosane in the reactor and C_{20}^{Final} was the final mass of *n*-eicosane in the reactor.

To determine the molecular weight distribution for residual PE, samples consisting of the residual polymer and catalyst mixture from the reactor were first allowed to dry at ambient conditions to remove low volatility liquids. An approximately 250 mg sample of the recovered solid (catalyst and residual PE) was then enclosed in packets of stainless steel (filtering) mesh and placed into 10 mL of 1,2,4-trichlorobenzene with 300 ppm (mg/kg) Irganox 1010 (CAS: 6683-19-8, BASF – North America, Florham, NJ, USA) as an antioxidant. The samples were heated with the solvent for 2 hours at 145 °C. The mesh packets were removed, and the solutions were then transferred to the appropriate chromatography vials for analysis. This recovered sample and aliquots of NIST SRM-1475A,[1] a linear PE substrate (certified M_w = 52,000 g/mol ± 2,000 g/mol), were analyzed by high temperature size exclusion chromatography (HT-SEC) using a Tosoh HT-EcoSEC instrument (Tosoh – North America, Grove City, OH) with differential refractive index (RI) detection. Separations were conducted at 135 °C using 1,2,4-trichlorobenzene (CAS: 120-82-1, Sigma Aldrich, St. Louis, MO, USA) as the eluent, with 300 mg/kg Irganox 1010 added as antioxidant to the solvent reservoir. Five μL of dodecane (CAS: 112-40-3, Sigma Aldrich, St. Louis, MO, USA) was added to each vial as a flow rate marker. The stationary phase was a set of 3 Tosoh HT columns (2 Tosoh TSKgel GMHHR-H (S) HT2, 13 μm mixed bed, 7.8 mm ID × 30 cm columns and 1 Tosoh TSKgel GMHHR-H (20) HT2, 20 μm, 7.8 mm ID × 30 cm column with an exclusion limit ≈ 4 × 10^8 g/mol). For the Tosoh instrument, narrow dispersity polystyrene standards were used for calibration and were converted to the PE equivalent using Mark-Houwink parameters for polystyrene and PE. The uncertainty in the molar masses obtained by this measurement is ±1.5%. All injections were done at least three times, and the reported error on all measurements is one standard deviation of the mean.

Catalyst Characterization

The elemental content of the catalysts was analyzed via inductively coupled plasma - optical emission spectrometry (ICP-OES) (Agilent 5110 ICP-OES, Agilent Technologies, Santa Clara, CA, USA). Initially,

approximately 25 mg of each catalyst was weighed out and dissolved in 10 mL of concentrated acid mixture (HNO_3 for Re_2O_7/γ-Al_2O_3 or 3:1 HNO_3:HCl for Pt/γ-Al_2O_3 and SnPt/γ-Al_2O_3. This mixture was heated in a Teflon vessel at 200 °C for 30 minutes in a microwave digestion system (CEM MARS5) operating at 1600 W. The digestate was filtered and diluted to 50 mL with DI water. A 5 mL aliquot of this solution was then combined with an additional 10 mL of the corresponding concentrated acid mixture and diluted again to 50 mL with DI water to produce a sample solution with appropriate concentrations and consistent sample matrix for ICP-OES analysis. Calibration standards of the relevant elements (Al, Pt, Re, Sn) were made at 1, 5, 10, 20, and 40 ppm in the same dilute acid matrix. Elemental concentrations were quantified after ICP-OES analysis using the following characteristic emission peaks: 396.152 nm (Al), 214.424 nm (Pt), 221.427 nm (Re), 235.485 nm (Sn).
Surface area was measured with nitrogen adsorption using the multipoint Brunauer-Emmett-Teller (BET) method performed with a Quantichrome Instruments (Boynton Beach, FL, USA) Quadrasorb. Approximately 0.1 g sample was degassed in UHP He at 200 °C for 16 h, then allowed to cool to ambient prior to collecting an adsorption and desorption isotherm at 77K with a 30% N_2 balance He carrier gas.

The active metal surfaces of the samples were probed with CO chemisorption on an Autosorb-1 by Quantichrome Instruments (Boynton Beach, FL, USA). Approximately 0.1 g of sample was packed with quartz wool into the sample tube. The sample was initially heated to 150 °C under N_2 and held at temperature for 60 minutes The flow was then changed to H_2 and the sample was heated to 200 °C and held at temperature for 120 min. The sample tube was then evacuated for 120 min, after which the furnace was cooled to the analysis temperature. Analysis was conducted under CO at 40 °C with a 16-point analysis and a 1 minutes thermal equilibration time.

Scanning transmission electron microscopy (STEM) was performed by first dispersing the catalyst particles onto ultrathin carbon film on lacey carbon support film transmission electron microscopy (TEM) grids purchased from Ted Pella (Redding, CA, USA). The TEM samples were examined in a Field Electron and Ion Company **(**FEI, Hillsboro, OR, USA) Tecnai F20 UltraTwin field-emitting-gun (FEG) scanning transmission electron microscope (STEM) operated at 200 kV. Energy dispersive X-ray spectroscopy (EDS) was performed using an EDAX (Mahwah, NJ, USA) Octane T Optima windowless Si drift detector (SDD) EDS system and processed using EDAX TEAM software.

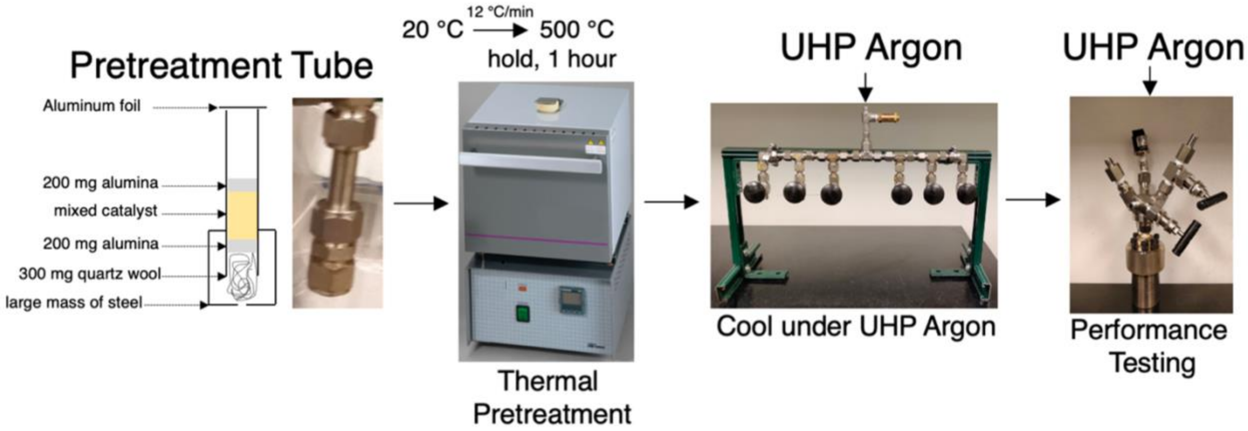

Figure S1. Pretreatment process. Custom ½" stainless steel tubes were loaded with 300 mg of quartz wool, followed by 200 mg of γ-Al_2O_3, catalyst, and 200 mg of γ-Al_2O_3. The tubes were then covered in perforated aluminum foil and heated in a muffle furnace and heated at 12 °C/min to 500 °C and held for 1 hour. Tubes were then rapidly transferred to a custom inerting reactor, allowing each tube to cool under UHP argon for 15 min. Tubes were then emptied into 75 mL Parr reactors, which were flushed with UHP argon during addition, and the sealing process. Reactors were then loaded onto a 5000 series 6-Well Parr Instruments reactor system.

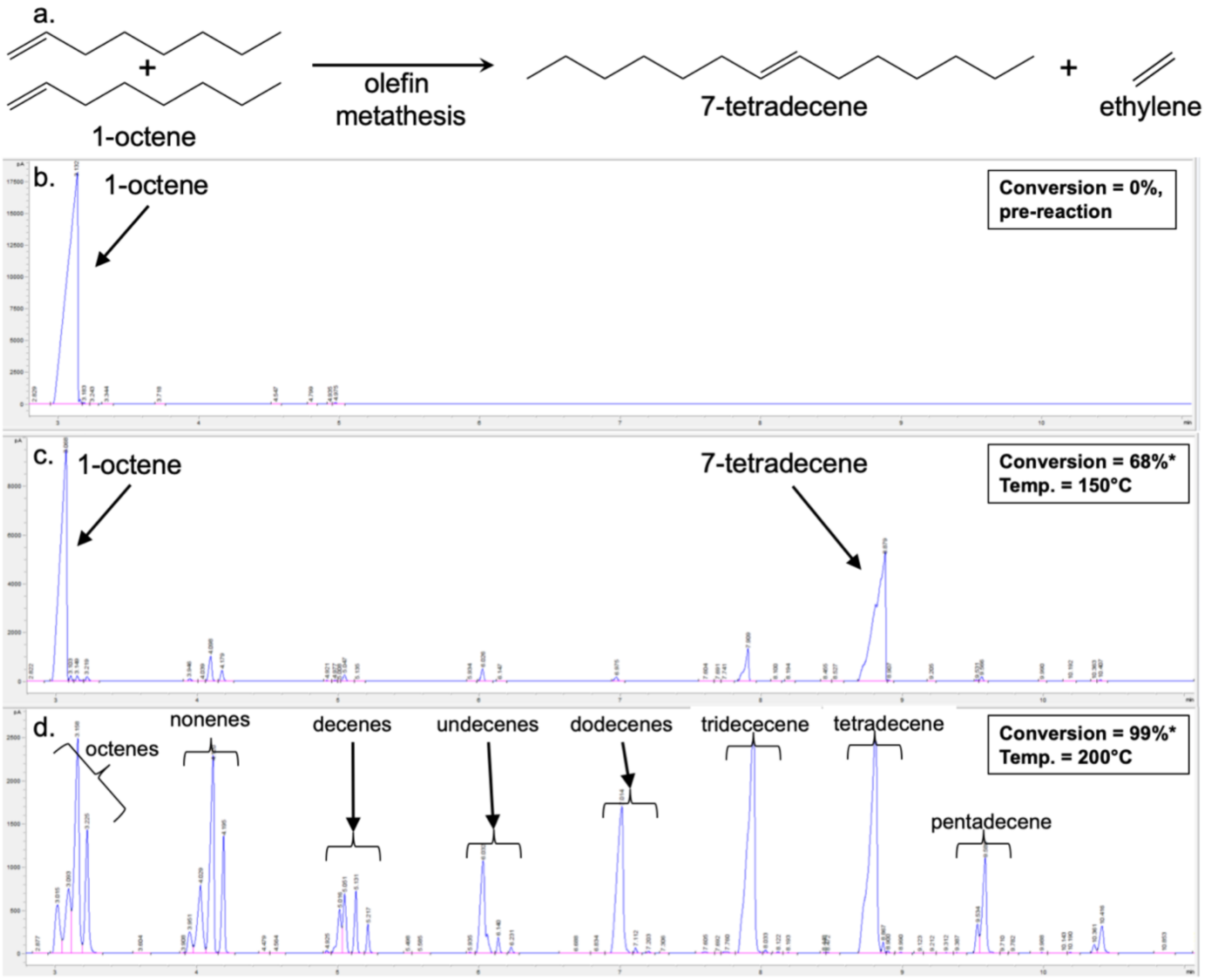

Figure S2. The pretreatment method (Figure S1) was developed using (a.) the coupling of 1-octene in *n*-pentane to 7-tetradecane and ethylene. Gas chromatography with a flame ionization detector (GC-FID) was utilized to monitor the residual reactant and products. (b) Chromatogram of the pre-reaction mixture of 10% (g/g) 1-octene in *n*-pentane. (c) Chromatogram of the residual reactant and products from a reaction of 250 mg of $Re_2O_7/\gamma\text{-}Al_2O_3$ at 150 °C for 1.5 hours. (d) Chromatogram of the residual reactant and products from a reaction of 250 mg of $Re_2O_7/\gamma\text{-}Al_2O_3$ at 200 °C for 1.5 hours. Liquid samples were analyzed by injection on a 6850 Agilent Gas Chromatograph with a HP-5MS column (30 m x 0.250 mm x 0.25 µm) with a temperature ramp method 50 °C (hold 1 min), 15 °C/min to 180 °C (hold 1 min), ramp 20 °C/min to 325 °C (hold 6 min), with a FID. Product identities were confirmed with GC-MS. *Estimated conversion (FIDarea/FIDarea).

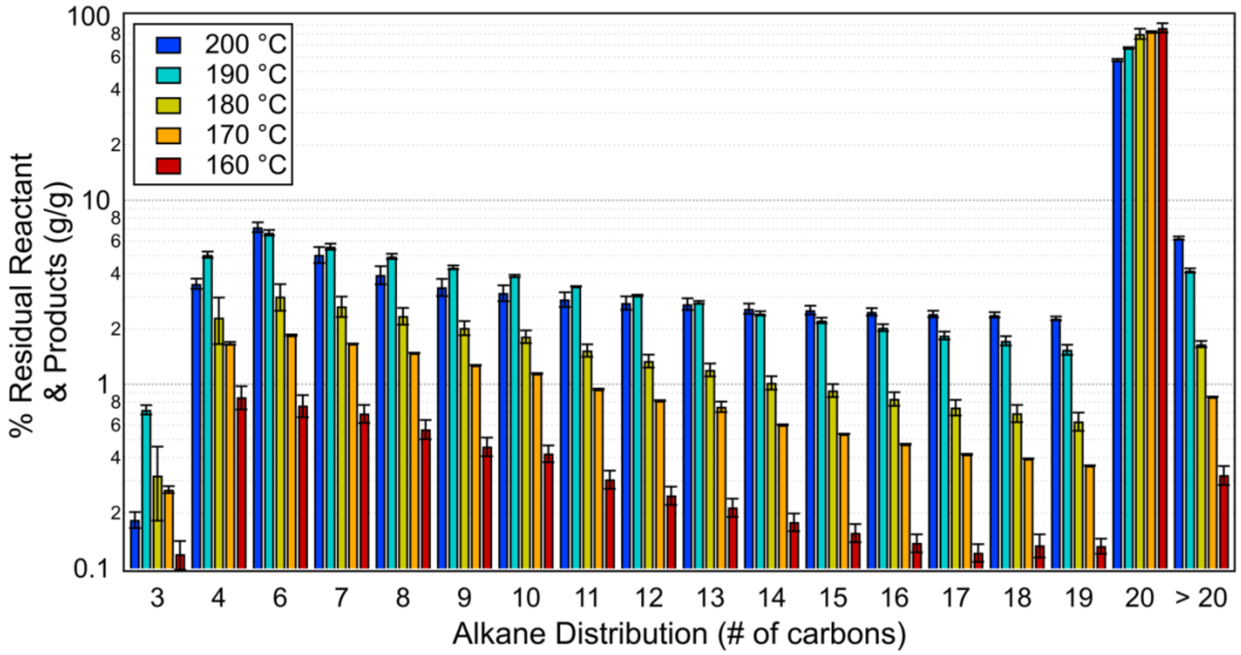

Figure S3. Residual reactant and product distribution from varying reaction temperatures for the alkane rearrangement of 5% (g/g) *n*-eicosane (C_{20}) in *n*-pentane with 500 mg Re_2O_7/γ-Al_2O_3 and 500 mg SnPt/γ-Al_2O_3 at 160 °C, 170 °C, 180 °C, 190 °C, and 200 °C for 15 hours. Data are shown as reaction duplicates, with error bars representing ± half the range of two measurements.

Table S1. BET surface area, chemisorption area, and elemental analysis of the catalysts used in this study. Post reaction catalysts were isolated after 15 hour reaction at 200°C with *n*-pentane only.

Catalyst	BET Surface Area	CO Monolayer Uptake	Elemental Analysis		
			Pt	Re	Sn
	m^2/g	µmol/g	% (g/g)	% (g/g)	% (g/g)
Re_2O_7/γ-Al_2O_3	194.9	0.4	0.1%	9.5%	0.0%
5% Pt/γ-Al_2O_3	94.5	82.0	5.7%	0.0%	0.0%
0.8% Pt/γ-Al_2O_3	206.0	21.3	0.8%	0.0%	0.0%
SnPt/γ-Al_2O_3	192.5	39.7	0.9%	0.0%	2.0%
Re_2O_7 on SnPt/γ-Al_2O_3	143.5	50.2	0.8%	7.9%	1.4%
SnPt on Re_2O_7/γ-Al_2O_3	145.3	55.5	0.9%	7.7%	1.5%
1:1 mixture of SnPt/γ-Al_2O_3 & Re_2O_7/γ-Al_2O_3, post-reaction	-	10.2	0.3%	3.0%	0.6%

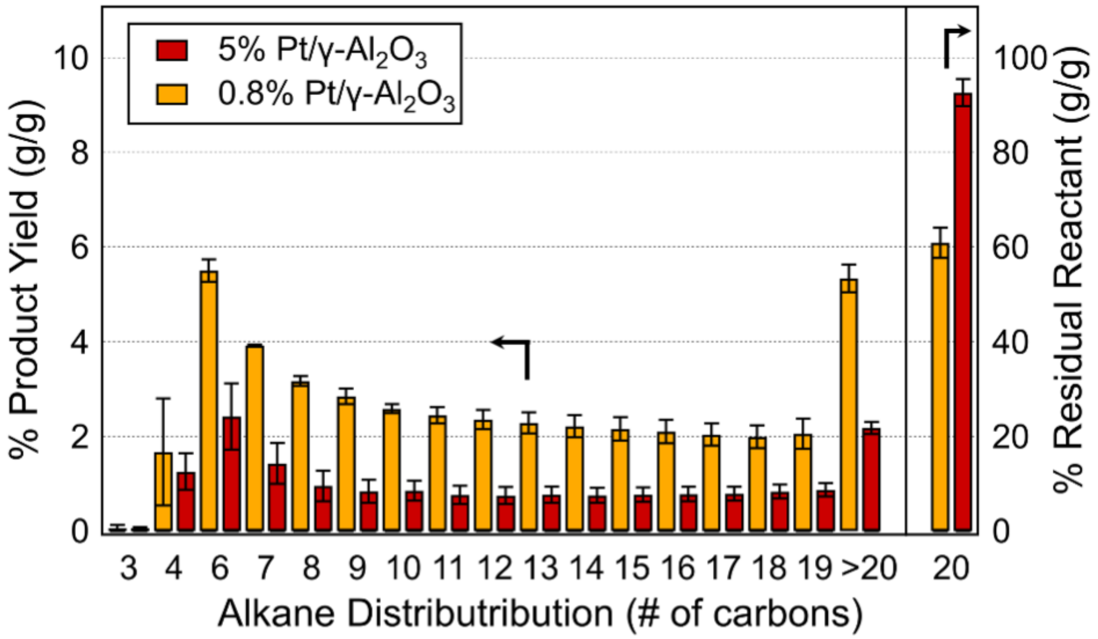

Figure S4. Comparison of the product yield (g/g) and *n*-eicosane conversion (g/g) for 5% (g/g) *n*-eicosane in *n*-pentane using two different dehydrogenation catalysts, either 500 mg of 5% Pt/γ-Al_2O_3 (commercial) or 500 mg 0.8% Pt/γ-Al_2O_3 (synthesized), in an 1:1 physical mixture with 500 mg of Re_2O_7/ γ-Al_2O_3. The reaction was performed at 200°C for a duration of 15 hours. Data are shown as the average of reaction duplicates with error bars representing ± half the range of two measurements.

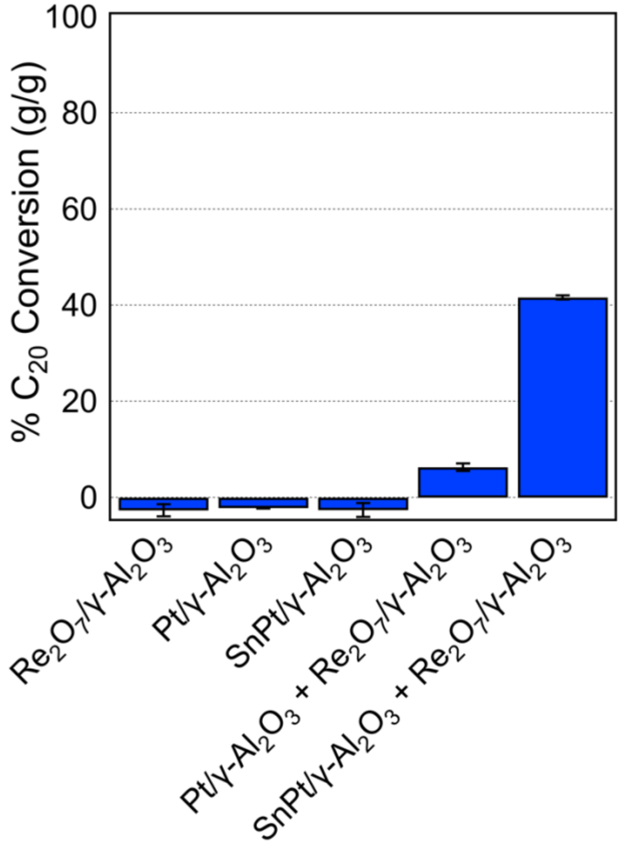

Figure S5. Conversion of 5% (g/g) *n*-eicosane in *n*-pentane using 500 mg of Re_2O_7/ γ-Al_2O_3, 500 mg Pt/γ-Al_2O_3, 500 mg SnPt/γ-Al_2O_3, Pt/γ-Al_2O_3 & 500 mg Re_2O_7/γ-Al_2O_3, or 500 mg SnPt/ γ-Al_2O_3 & 500 mg Re_2O_7/γ-Al_2O_3 at 200 °C for 15 hours. Data are shown as the average of reaction duplicates with error bars representing ± half the range of two measurements.

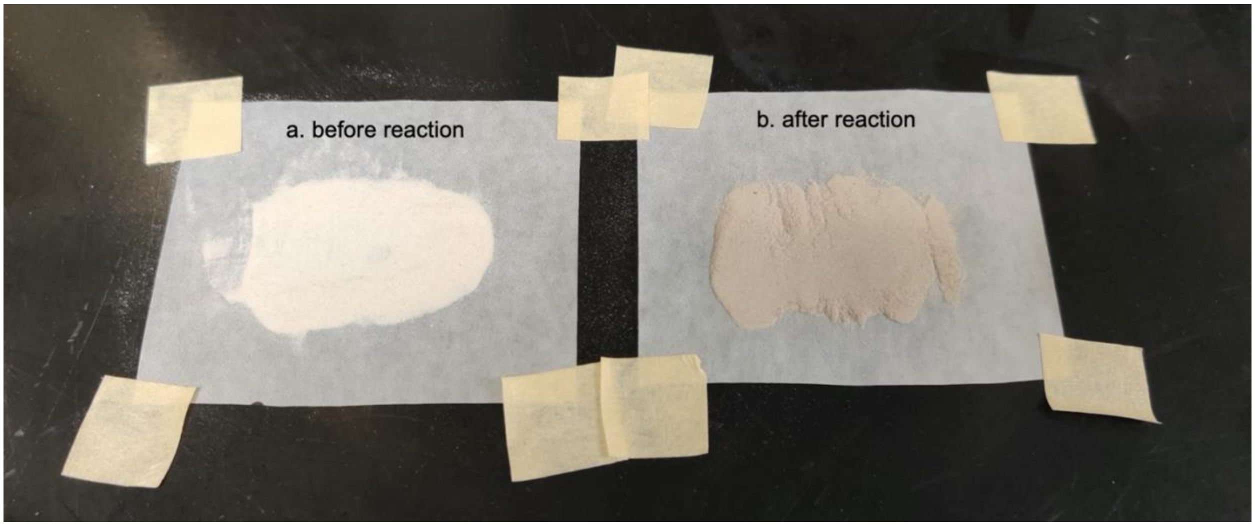

Figure S6. Image of a 1:1 physical mixture of SnPt/γ-Al_2O_3 and Re_2O_7/γ-Al_2O_3 (a.) before and (b.) after reaction with *n*-pentane only for a duration of 15 hours at 200°C.

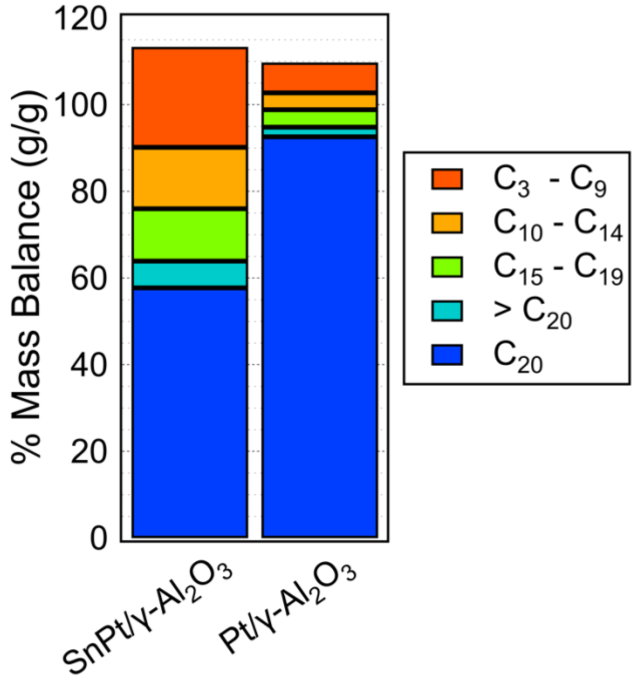

Figure S7. Mass balance of the products and residual C_{20} substrate using 500 mg Re_2O_7/γ-Al_2O_3 and either 500 mg SnPt/γ-Al_2O_3 or Pt/γ-Al_2O_3. The reaction was run at 200 °C for 15 hours with 5% (g/g) *n*-eicosane (C_{20}) in *n*-pentane. Data shown as the average of reaction duplicates.

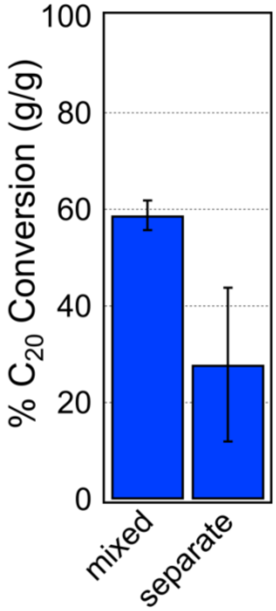

Figure S8. Conversion of 5% (g/g) *n*-eicosane (C_{20}) in *n*-pentane with 750 mg of SnPt/γ-Al_2O_3 and 750 mg of Re_2O_7/γ-Al_2O_3 at 200 °C for 15 hours. Pretreatment was performed as a physical mixture of both catalysts in a single pretreatment tube (mixed) or in two separate pretreatment tubes (separate). Data are shown as an average of reaction triplicate (mixed) and quadruplicate (separate) with error bars shown as standard deviation.

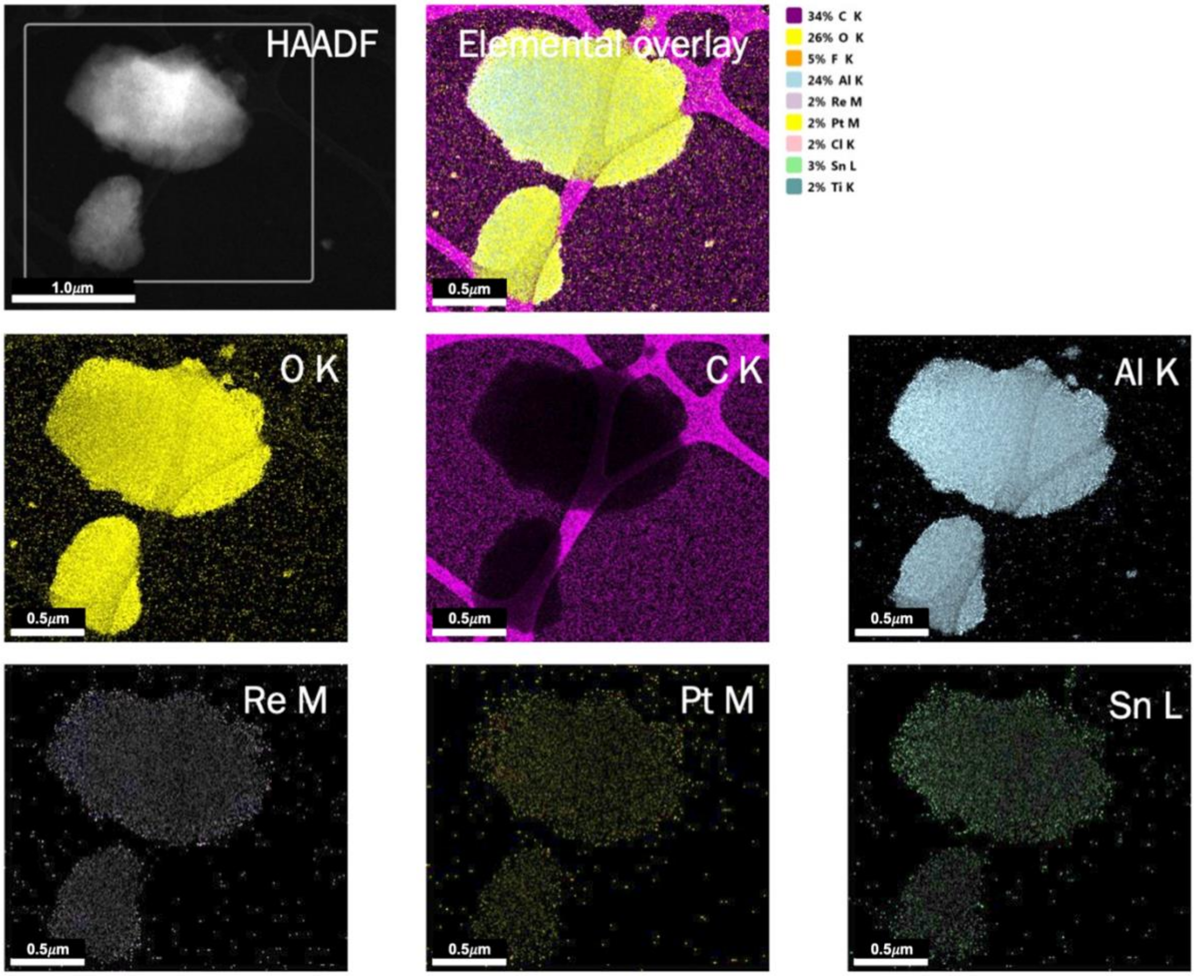

Figure S9. Scanning transmission electron microscopy with energy dispersive X-ray spectroscopy (STEM-EDS) atomic percent maps of a 1:1 physical mixture of Re_2O_7/γ-Al_2O_3 and SnPt/γ-Al_2O_3, after the typical pretreatment procedure (Figure S1). When pretreated separately, no particles were found to have Re, Pt, and Sn. However, after pretreatment, six out of eight particles analyzed contained Re, Pt, and Sn on a single support, while the remaining two contained Sn and Re.

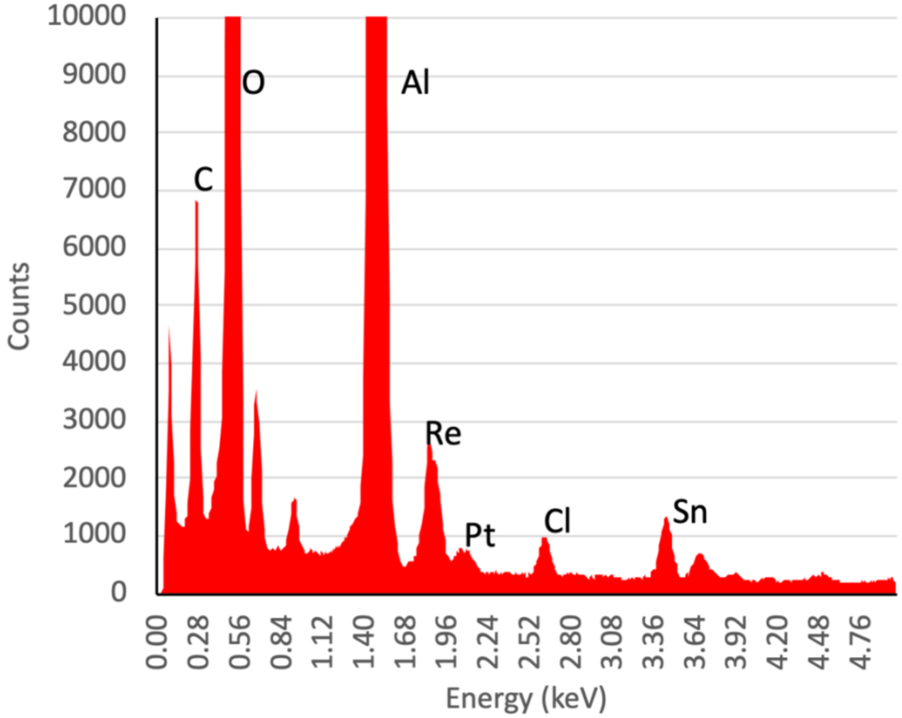

Figure S10. Energy Dispersive X-ray Spectra for a 1:1 physical mixture of Re_2O_7/γ-Al_2O_3 and SnPt/γ-Al_2O_3, after the typical pretreatment procedure (Figure S1), C = Carbon, O = Oxygen, Al = Aluminum, Re = Rhenium, Pt = platinum, Cl = chlorine, and Sn = tin. The powder samples were dispersed on an ultrathin carbon film on lacey carbon support film TEM grids.

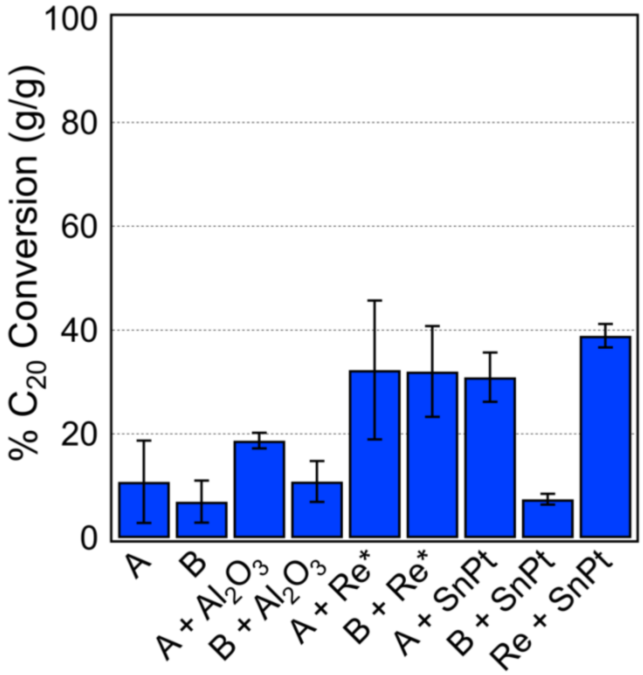

Figure S11. Conversion of 5% (g/g) *n*-eicosane in *n*-pentane performed at 200 °C for a duration of 15 hours with the following catalysts, A = 500 mg of Re_2O_7 deposited on SnPt/γ-Al_2O_3, B = 500 mg of SnPt/γ-Al_2O_3 deposited on Re_2O_7/γ-Al_2O_3, Al_2O_3= 500 mg of additional γ-Al_2O_3, Re = 500 mg of additional Re_2O_7/γ-Al_2O_3, and SnPt = 500 mg of additional SnPt/γ-Al_2O_3. Reactions were run in duplicate, with the error bars shown as the half range; *reactions were run in triplicate, with the error shown as standard deviation.

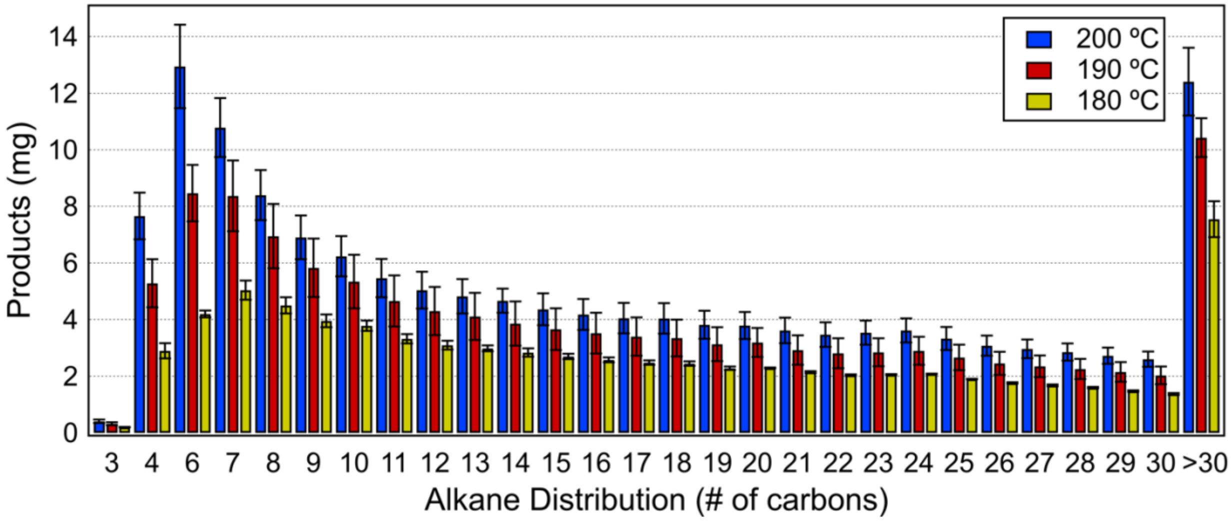

Figure S12. Distribution of linear alkane products after depolymerization of a 250 mg of SRM-1475 feedstock (PE, M_w = 54.1 ± 2 kDa) in *n*-pentane, with a physical mixture of two catalysts, 500 mg Re_2O_7/γ-Al_2O_3 and 500 mg SnPt/γ-Al_2O_3. Reactions were performed at reaction temperatures of 180 °C, 190 °C, or 200 °C for 15 hours. Data are presented as an average of reaction duplicates with error bars shown as ± half the range of two measurements.

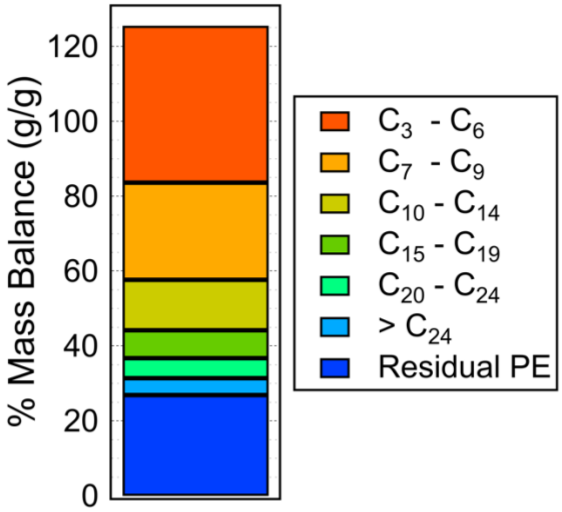

Figure S13. Mass balance of the products and estimated PE recovered. Estimated PE recovery was based on the reduction in molecular weight and the starting mass of feedstock. Reaction was run with 500 mg SnPt/γ-Al_2O_3 and 500 mg Re_2O_7/γ-Al_2O_3, at 200 °C for a duration of 15 hours with 130 mg of SRM-1475 feedstock (PE, M_w = 54.1 ± 2 kDa). Data are shown as the average of reaction triplicates.

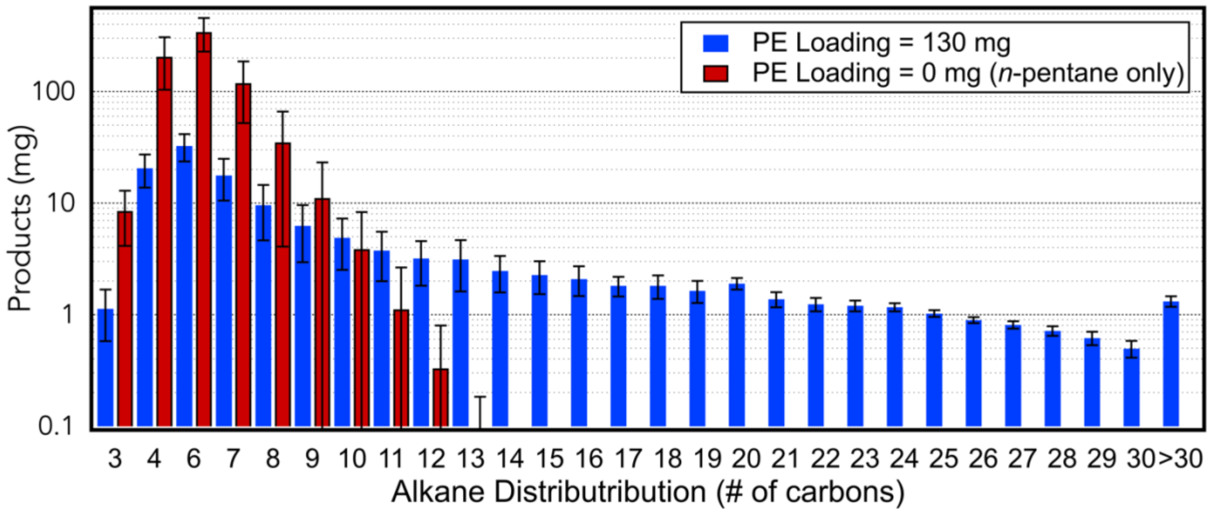

Figure S14. Distribution of linear alkane products after depolymerization of a 130 mg of a SRM-1475 feedstock (PE, M_w = 54.1 ± 2 kDa) in *n*-pentane or *n*-pentane only, with a physical mixture of two catalysts, 750 mg $Re_2O_7/\gamma\text{-}Al_2O_3$ and 750 mg $SnPt/\gamma\text{-}Al_2O_3$. Reactions were performed at 200 °C for a duration of 15 hours. Data is presented as an average of reaction triplicates with error bars shown as standard deviation.

REFERENCES

(1) Hoeve, C. A.; Wagner, H. I.; Verdier, P. H. The Characterization of Linear Polyethylene SRM 1475. I. Introduction. *J. Res. Natl. Bur. Stand. Sect. A Phys. Chem.* **1972**, *76A* (2), 163. https://doi.org/10.6028/jres.076a.023.
